# Localization Free Super-Resolution Microbubble Velocimetry Using a Long Short-Term Memory Neural Network

**DOI:** 10.1109/TMI.2023.3251197

**Published:** 2023-08-01

**Authors:** Xi Chen, Matthew R. Lowerison, Zhijie Dong, Nathiya Vaithiyalingam Chandra Sekaran, Daniel A. Llano, Pengfei Song

**Affiliations:** Department of Electrical and Computer Engineering, Beckman Institute for Advanced Science and Technology, University of Illinois Urbana–Champaign, Urbana, IL 61801 USA; Department of Electrical and Computer Engineering, Beckman Institute for Advanced Science and Technology, University of Illinois Urbana–Champaign, Urbana, IL 61801 USA; Department of Electrical and Computer Engineering, Beckman Institute for Advanced Science and Technology, University of Illinois Urbana–Champaign, Urbana, IL 61801 USA; School of Molecular and Cellular Biology, Beckman Institute for Advanced Science and Technology, University of Illinois Urbana–Champaign, Urbana, IL 61801 USA; School of Molecular and Cellular Biology, Beckman Institute for Advanced Science and Technology, University of Illinois Urbana–Champaign, Urbana, IL 61801 USA; Department of Electrical and Computer Engineering, the Department of Bioengineering, Beckman Institute for Advanced Science and Technology, University of Illinois Urbana–Champaign, Urbana, IL 61801 USA

**Keywords:** Contrast ultrasound, deep-learning, microbubble, super-resolution imaging, ultrafast ultrasound

## Abstract

Ultrasound localization microscopy is a super-resolution imaging technique that exploits the unique characteristics of contrast microbubbles to side-step the fundamental trade-off between imaging resolution and penetration depth. However, the conventional reconstruction technique is confined to low microbubble concentrations to avoid localization and tracking errors. Several research groups have introduced sparsity- and deep learning-based approaches to overcome this constraint to extract useful vascular structural information from overlapping microbubble signals, but these solutions have not been demonstrated to produce blood flow velocity maps of the microcirculation. Here, we introduce Deep-SMV, a localization free super-resolution microbubble velocimetry technique, based on a long short-term memory neural network, that provides high imaging speed and robustness to high microbubble concentrations, and directly outputs blood velocity measurements at a super-resolution. Deep-SMV is trained efficiently using microbubble flow simulation on real in vivo vascular data and demonstrates real-time velocity map reconstruction suitable for functional vascular imaging and pulsatility mapping at super-resolution. The technique is successfully applied to a wide variety of imaging scenarios, include flow channel phantoms, chicken embryo chorioallantoic membranes, and mouse brain imaging. An implementation of Deep-SMV is openly available at https://github.com/chenxiptz/SR_microvessel_velocimetry, with two pre-trained models available at https://doi.org/10.7910/DVN/SECUFD.

## I. INTRODUCTION

ULTRASOUND localization microscopy (ULM) is a super-resolution imaging technique that relies on the localization and tracking of intravascular microbubble (MB) contrast agents to reconstruct microvasculature. It has been demonstrated by multiple research groups that MBs can act as acoustic point sources that can be localized at a sub-diffraction precision, achieving micrometer-scale vascular fidelity at clinical frequencies without sacrificing imaging depth [1], [2], [3], [4], [5]. Furthermore, a unique feature of ULM is the ability to measure the blood flow velocity of the microvasculature, which serves as a sensitive biomarker for numerous physiological and pathological states. The ULM MB localization protocol side-steps a fundamental trade-off between imaging resolution, which is dictated by wavelength, and imaging penetration depth, which is limited by attenuation. However, ULM introduces a new consideration between MB localization precision and acquisition time. High-fidelity MB localization requires non-overlapping MB signals; but the dilution of MBs used to achieve spatially sparse signals necessitates a long imaging duration to ensure the full perfusion of all patent vasculature [6], [7]. This exasperates the pragmatic challenges associated with super-resolution vascular reconstruction, including sources of tissue motion and time-varying changes in vascular flow, which severely limit the clinical application of the technology. High concentration MB injections will perfuse through more of the vasculature over the same duration, permitting shorter imaging acquisition windows but at the expense of MB localization precision with increased spurious events and erroneously reconstructed features. Rectifying these two contradictory requirements for ULM is an area of ongoing and active research for super-resolution ultrasound.

Several ULM approaches have been specifically introduced to handle overlapping MB signals from high-concentration contrast injections. Sparse recovery strategies [8], [9], such as sparsity-based ultrasound super-resolution hemodynamic imaging (SUSHI), utilize a structurally sparse prior-representing vasculature to resolve overlapping MB point-spread functions (PSF). This localization-free approach improves imaging speed but cannot measure blood flow velocity. Compressive sensing algorithms have demonstrated the ability to resolve high density MB localizations [10], [11] and deconvolution-based approaches can iteratively shrink the imaging PSF, resulting in better localization accuracy for overlapping MB signals [12], [13]. These approaches improve localization accuracy and can generate high-fidelity microvascular density maps but sacrifice the ability to track individual flowing MB trajectories. Super-resolution with acoustically activated nanodroplets has demonstrated extremely short data acquisition times in vivo preclinical studies [14], [15]. Deep-learning (DL) solutions are also gaining popularity for ULM imaging, with numerous classes of neural network architecture and training data generation showing promise in improving localization accuracy, particularly for high MB concentrations, and for accelerating the time-consuming ULM processing pipeline. Specifically, van Sloun et al. [16] and Liu et al. [17] used neural network architectures to extract point target locations representing MB centroids from B-mode images in the spatial domain. Lok et al. [18] and Milecki et al. [19] used spatiotemporal data and a fully convolutional network to improve localization confidence. These proposed DL solutions directly generate super-resolved microvessel maps but, as with the above approaches, have not been demonstrated to produce blood flow velocity maps. Thus, despite these promising results, a true high-speed super-resolution technology that can handle high concentration MB injections and offer all the features of conventional ULM (that is, microvascular structure and velocity) has not been previously reported. In principle, frame-to-frame pairing and tracking can be applied after high-concentration localization strategies, such as in [20] and [21], to supply velocity information. However, such data association solutions are challenging for high density localization datasets.

Here we introduce DL-based super-resolution microvessel velocimetry (Deep-SMV), a localization free approach that provides high imaging speed and robustness to high MB concentrations, and directly outputs blood velocity measurements at a super-resolution without explicit data association for MB tracking. Deep-SMV leverages the rich spatiotemporal information embedded in ultrasound MB imaging data with a convolutional neural network including long short-term memory (LSTM) blocks to provide both structural super-resolution imaging and time-resolved velocimetry of tissue vasculature, permitting super-resolution pulsatility mapping. LSTM is a type of recurrent neural network (RNN) architecture [22], [23] which is typically characterized as neural networks with “feedback loop” mechanisms that use previous output as input and maintain internal hidden states. Compared to using feedforward networks for temporal inference with an additional dimension representing the temporal dimension, RNNs, designed specifically to handle temporal sequences, are usually more efficient in learning temporal features. By keeping internal hidden states that are constantly updated with new input, RNNs retain memory of past inputs that affects the processing of the current input. The length of the historical events that an RNN keeps track of can be potentially infinite. LSTM addresses the vanishing gradient problem in classical RNNs by introducing gates to modulate information flow within the network. RNNs and LSTM have been successfully applied to various tasks such as machine translation, object tracking and motion prediction [24], [25], [26]. In this implementation, Deep-SMV was trained to recognize diffraction-limited MB signals, ranging from spatially sparse to heavily overlapping conditions, to generate super-resolved microvascular structural and velocity maps. By eliminating the need for the highly inefficient process of MB localization and tracking, Deep-SMV provides effective and fast super-resolution imaging by more efficiently exploiting the spatiotemporal information from the input data. We first evaluate the performance of Deep-SMV using in vitro flow channel phantoms and then on in vivo ultrasound MB data taken from the chorioallantoic membrane (CAM) of chicken embryos, which provides an optical reference standard. We then demonstrate Deep-SMV’s ability to handle more complex microvascular dynamics by applying the technique to super-resolution velocimetry of the mouse brain.

## II. Methods

### A. Deep-SMV Architecture and Design

**Fig. 1.**
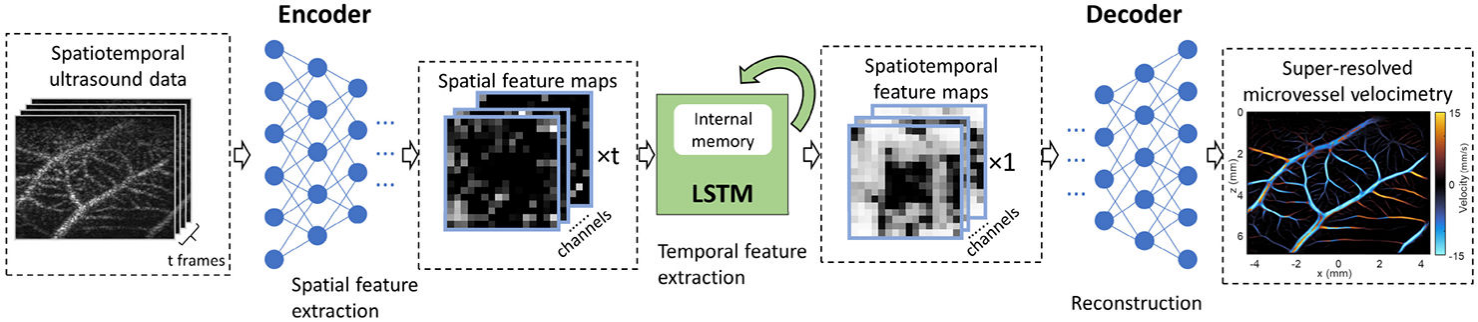
Deep-SMV data workflow. The Deep-SMV network takes a spatiotemporal (2D spatial + time) input of ultrasound data, passes this through encoder blocks to extract spatial features, uses LSTM blocks to extract temporal features, and then reconstructs a super-resolved velocimetry map from the spatiotemporal features via decoder blocks.

**Fig. 2.**
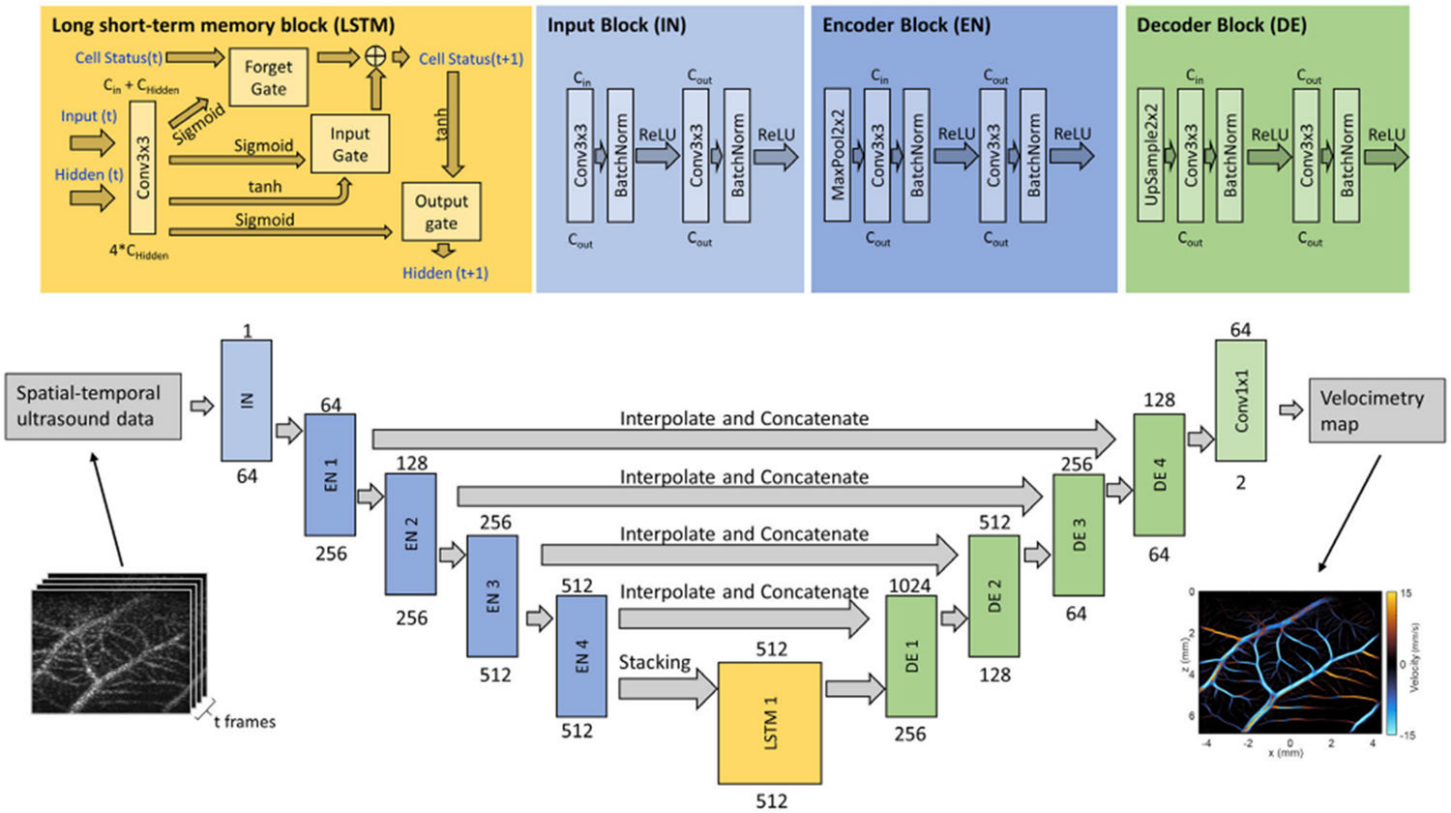
Deep neural network architecture for Deep-SMV. The main network design is a classic UNet structure with long short-term memory (LSTM) blocks in the bottleneck layers to provide flow velocity measurements, with input consisting of spatial-temporal (2D spatial + time) ultrasound data and output of super-resolution velocity and structure maps. The LSTM, input, encoder, and decoder block designs are provided in detail at the top of the figure. The text above each block indicates the input channel size to the unit, and the text under each block indicates the output channel size from the unit.

Our NN model adopts the convolutional LSTM-UNet structure, where the bottleneck layers of the classical UNet were replaced by LSTM layers (Fig. 1, Fig. 2). The feature extraction path of the network starts with an input block that contains two convolution layers with 3*3 kernel size, each followed by a batch normalization layer and a Rectified Linear Unit (ReLU) activation function. The encoder block contains a 2*2 max pooling layer, two 3*3 convolution layers each followed by a batch normalization layer and ReLU activation function. Each encoder block reduces all spatial dimensions of its input by a factor of two and expands the feature dimension by a factor of two. Each frame of 2-D spatial input will be fed into the feature extraction blocks separately. The resulting feature maps were concatenated along the temporal dimension before being fed to the convolutional-LSTM layers as a feature map sequence. This approach allows for the Deep-SMV network to directly train for the output of blood flow velocity from the raw spatiotemporal features, without explicitly relying on inefficient MB localization and tracking processes. This permits the network to use high concentration and overlapping MB signals more efficiently for large vascular lumens while retaining high fidelity for microvascular reconstruction.

The convolutional-LSTM block takes a new input and the previous hidden state output as it inputs, while maintaining an internal cell status variable. The inputs will first go through a 3*3 convolution layer. The convolution layer output will be split along the channel dimension into four parts, where three will go through a sigmoid activation function and operate as the forget gate, the input gate, and the output gate, respectively. A hyperbolic tangent (tanh) function will be applied to the remaining part. The forget gate determines which part of the previous cell status will be discarded. The input gate determines whether parts of the new input contribute to updating the cell status. A tanh activation function will be applied to the new cell status. Finally, the output gate determines how the cell status propagates to the output. The convolutional LSTM block will reduce the input sequence along the temporal dimension.

The decoder block is constructed similarly to the encoder block, with the max pooling layer replaced by spatial upsampling layer. Each decoder block will expand the spatial dimension of its input by 2 and reduce the channel dimension by 2. The output block is a 3*3 convolution layer with output channel size of 2. The final output of the network is a predicted blood flow velocity map with the same spatial dimension as the input data.

We used mean squared error (MSE) as the loss function for training the NN, defined as:

(1) $$MSE\;(\hat{y},y)=\frac{1}{n}\sum_{i=1}^{n}\;({\hat{y}}_{i}-y_{i}{)}^{2},$$

### B. CAM Optical and Mouse Brain ULM Image-Based Training Data Simulation

The training dataset of our NN model was simulated using CAM optical images, as well as conventional mouse brain ULM results to serve as a vessel structure template.

#### 1) CAM Optical Image Acquisition:

**Fig. 3.**
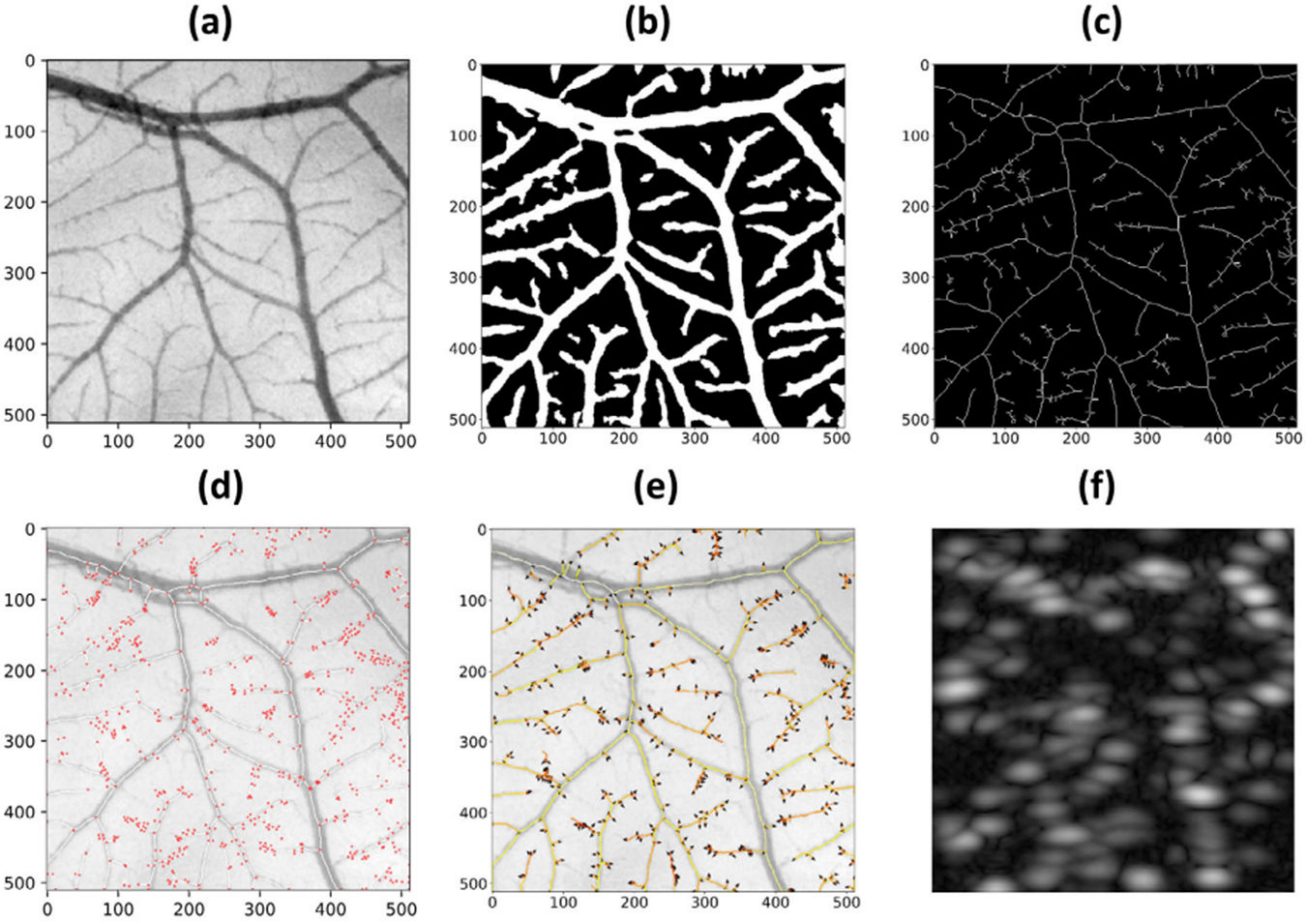
CAM-based MB flow simulation procedure. (a) Grayscale image of the green channel pixel intensity of a 512-by-512 pixels region in a CAM optical image. (b) Binary segmentation result of (a) by applying adaptive thresholding. (c) Skeleton of (b) obtained by applying medial axis skeletonization on (b). (d) Undirected graph constructed from the skeleton (b), displayed on top of the original CAM image. Lines represent edges and dots represent vertices. (e) Final directed vessel graph with arrows indicating flow direction on each vessel segment. (f) Example frame of Field-II ultrasound simulation from MB flow simulation.

Optical imaging was performed using a Nikon SMZ800N stereomicroscope (Nikon, Tokyo, Japan) with a Plan Apo 1x objective at 1x zoom, captured with a DS-Fi3 microscope camera, and saved as.nd2 format. These were later exported as RGB .tiff files for external analysis (Fig. 3). The optical imaging field of view was 11.01 mm × 15.48 mm yielding 2048-by-2880 pixel images.

#### 2) CAM Vessel Flow Dynamics Model:

A binary vessel map of each CAM vasculature (Fig. 3a) was segmented by applying the *threshold_adaptive* function available in the scikit-image python package [27] to the green channel of each acquired optical image (Fig. 3a) as it provides the best contrast for blood vessels. Non-overlapping regions of 256-by-256 pixels were sampled from 35 2048-by-2880 pixel binarized CAM optical images, resulting in 3080 binarized ROIs, from which 1356 regions with acceptable segmentation performance were manually selected for further processing.

Each binary vessel map was skeletonized using the medial_axis and *skeletonize* function, also available in the scikit-image package. The medial_axis and *skeletonize* function returns the skeletonized image (Fig. 3c), as well as the distance transform map which can be used as an estimation of local width of the vasculature. The skeleton image is converted to an undirected graph G = {V, E}, where E is the set of edges that represents vessel segments, and V is the set of vertices that represents junctions of the vessel segments (Fig. 3d). The graph is stored as a NetworkX [28] undirected graph. Conversion from skeleton image to NetworkX graph is achieved using the *sknw* function available in the ImagePy image processing framework [29].

The undirected vessel graph was then separated into a set of subgraphs, where each subgraph is a connected component of G. A spanning tree (a connected acyclic subgraph that covers all vertices in the original graph) was computed for each subgraph using the breadth-first search (BFS) algorithm starting from a source point defined as the degree-1 vertex with the largest local width in the corresponding distance map from the skeletonization step. Directions of the edges in the tree were assigned to start from the vertex that appeared earlier in the BFS traversal and point to the vertex that appeared later in the BFS traversal. The final directional vessel graph (Fig. 3e) is a forest that contains all the directional spanning trees obtained from each connected component of the undirected vessel graph.

#### 3) Calculating Reference Velocity:

We used the CAM ULM result as a reference to calculate a distribution of blood flow velocity on the central line of vessels for a given radius. We apply adaptive thresholding on the ULM velocity map to generate a binary vessel map, then perform medial axis skeletonization to obtain a local vessel radius estimation for each pixel on the medial axis of the vessel structure. All local radius-velocity pairs of pixels on the medial axis were sorted in ascending order of the local distances. The estimated reference velocity on the center line of a vessel with radius d was drawn from a normal distribution N(μ, $\sigma^{2}$), where μ is the average velocity of all radius-velocity pairs with radius within d±0.5 pixels range in the list of radius-velocity pairs, and σ^2^is the variance of the selected pairs.

#### 4) Mouse Brain ULM-Based Flow Dynamics Model:

To study the effect of tissue-specific vascular structures in the training dataset, in addition to CAM-based vessel flow model, we also generated a simulation training set of mouse brain vasculature. Since optical images of the brain were not available, we directly used the ULM reconstruction to construct the vessel models. 40 mouse brain ULM images (imaging field of view 7.17mm axial by 5.96mm lateral; λ/10 interpolation) were sampled as 256*256 pixels regions with a step-size of 128 pixels. The ULM images were generated from accumulated tracks of individual MBs, therefore will contain gaps and fragments even after long accumulation. For each sub-region, we apply *remove_small_holes* function from the skimage.morphology python package to fill the gaps and holes in ULM microvessel density map, followed by *remove_small_object* to clean up the remaining fragmented tracks. We then apply the same binarization-skeletonization procedure as described in the CAM-based simulation case and convert the cleaned-up vessel images into undirected graphs.

The undirected graphs were converted into directed vessel flow dynamics model with the same fashion as discussed in the CAM-based simulation case, except for velocity assignment. Here, we can directly use the corresponding mouse brain ULM velocity map. For each vessel segment, we calculate the average velocity of all the non-zero pixels in the ULM velocity map within the area of the segment and use it as the velocity along the center line of the vessel segment in the directed vessel flow graph.

#### 5) Simulating MB Motion:

Once the vessel flow dynamics model was constructed, we can use it to generate a sequence of MB locations that travel in the vessel network. Each MB was stored as a tuple ($e_{orig}$, $e_{curr}$, $d_{ax}$, $d_{lat}$, amp), where $e_{orig}$ is the vessel segment (edge) that it originated from, $e_{curr}$ is the edge that it is currently traveling along, $d_{ax}$ is the axial distance with respect to the current edge, $d_{lat}$ is the lateral distance with respect to the current edge, and *amp* is the amplitude of the MB. The spatial location of the MB with respect to the entire field-of-view (FOV) can be easily inferred from $e_{curr}$, $d_{ax}$ and $d_{lat}$. The MB location was updated using the following rules:

Initialization: place $n=\frac{a_{e}}{A}N$ MBs on each edge, where A is the total volume of all vessel segments, $a_{e}$ is the volume of the current vessel segment, estimated using the local radius at points on the edge $e,a_{e}=\sum_{i\in pointsone}\pi r_{i}^{2}$, and N is the total number of MBs to be placed in the vasculature. Set both $e_{orig}$ and $e_{curr}$ to be e. $d_{ax}$ and $d_{lat}$ were both randomly chosen so that the MB stays within the current vessel segment. Amp was randomly selected from a uniform distribution on (0, 1]. $e_{orig}$ and amp will remain unchanged until the MB is removed from the vessel network.

For each timestep, perform the following for each MBs:

- Calculate the axial velocity along the direction of the current vessel segment of each MB according to the Poiseuille flow condition:
(2) $$v_{ax}=v_{ref}\times (1-\frac{d_{lat}}{r}),$$

where $v_{ref}$ is the reference flow velocity on the centerline calculated using the method described in the previous section, and r is the local radius of the vessel segment.

- Calculate the displacement $v_{ax}\times \Delta t$ where Δt is the temporal resolution.
- If the displacement is smaller than the remaining length of the current edge:
  - Update $d_{ax}$ with the displacement.
  - Update $d_{lat}$ with a number randomly generated small distance so that the MB still stays within the vessel segment.
  - $e_{curr}$ remains unchanged.
- If the displacement is larger than the remaining length of the current edge:
  - If the current edge ends at a leaf vertex (i.e., no outgoing edge from the vertex), add a new MB to vessel segment $e_{orig}$ of the current MB, remove the old MB from the vessel network.
  - If the current edge has outgoing adjacent edges, the MB will be moved to one of the adjacent edges $e_{aj}$ with probability:
(3) $$\left(e_{aj}\right)=\frac{\pi r_{aj}^{2}}{\sum_{i\in out\;goingedges}\pi r_{i}^{2}},$$

where r is the local radius of the starting vertex of each edge. Subtract the remaining length on the current edge from the total displacement. Update $e_{curr}$ to $e_{aj}$. Repeat the steps above until the remaining displacement is less than the remaining length of the current edge. Then, update $d_{ax}$ and $d_{lat}$ with the same method in the previous step using the final remaining displacement.

Calculate the spatial coordinates of each updated MB using e, $d_{ax}$ and $d_{lat}$. Store the list of locations and amplitudes of each MB. After repeating the update steps for a desired duration of time, save the list of MB locations and amplitudes of each timestep as input to Field-II simulation.

#### 6) Field-II Simulation:

Ultrasound simulation of MB motion was performed in Field-II [30], [31] using the sequence of MB locations and amplitudes obtained in the previous simulated MB motion step. For each timestep, we generate one frame of ultrasound simulation of point scatterers specified by the corresponding list of locations and amplitudes in the sequence. The imaging sequence was configured according to the CAM ultrasound imaging experiment setting, with a high frequency linear array transducer, 20MHz center frequency, and 9-angle plane wave compounding. Detailed simulation parameters were summarized in Table I.

#### 7) Experimental PSF Simulation:

In addition to numerical simulation using ultrasound simulation software, we also explored the idea of incorporating experimental PSF in the simulation pipeline. Ultrasound images of isolated, slow flowing MBs were acquired by injecting heavily diluted (6 × 10^6^-fold dilution) MB solution into DI water and gently stirring to create movement. MB image patches were extracted using normalized cross correlation with an estimated Gaussian PSF template to create a bank of PSF samples. Simulation MB images were produced by convolution between the MB locations and amplitudes generated in Section II-B.5) and random selection of patches from the PSF bank. A same PSF patch was used for an individual MB throughout its existence in the vasculature, with slight rotation and distortion to emulate the appearance of realistic flowing MBs. Electronic noise was modeled as the magnitude to two complex normal random variables.

#### 8) Deep-SMV Training:

A total of 1300 training samples of 16 frames in the temporal dimension, and 256 * 256 pixels in the spatial dimension were fed to the model in mini-batches of 4 samples per batch. The model was trained with an Adam optimizer to minimize the MSE loss between the model output and the ground truth using back-propagation with a learning rate of 0.001. The ground truth contains two channels of the magnitudes in pixels/ms and angles of the flow velocity map. To avoid complications of training with negative values in the ground truth, the angle map which originally contains values in the range [−π,π] was scaled and shifted to the scale [0, 1]. The model was trained for a total of 100 epochs. Note that the downsample-upsample style structure of the model enables it to handle arbitrarily sized inputs. The network was applied to 16-frame sequences of full-sized images. The internal state of the LSTM layer was reset after processing each 16-frame block. The network trained using the CAM vessel model achieved a root mean squared error (RMSE) of velocity estimation of 0.94 mm/s (0.19 pixels per frame) for the simulation validation set. For the network trained using the mouse brain vessel model, the final RMSE on the validation set is 1.03 mm/s (0.21 pixels per frame).

### C. Ethics Approval

All procedures performed on mice at the University of Illinois Urbana-Champaign in this manuscript were approved by the Institutional Animal Care and Use Committee (IACUC). No IACUC approval was necessary to conduct the chicken embryo imaging, as per avian NIH PHS policy on avian embryos.

### D. Ultrasound Imaging

All flow phantom, chicken embryo, and mouse brain ultrasound data were acquired with a Vantage 256 system (Verasonics Inc., Kirkland, WA) with a L35-16vX high-frequency linear array transducer. Imaging was performed with a center frequency of 20 MHz, using 9-angle plane wave compounding (1-degree increments) with a post-compounding frame rate of 1,000 Hz. Ultrasound data was saved as in-phase quadrature (IQ) datasets of 1,600 frames each for external processing.

### E. Microbubble Flow Phantom

**Fig. 4.**
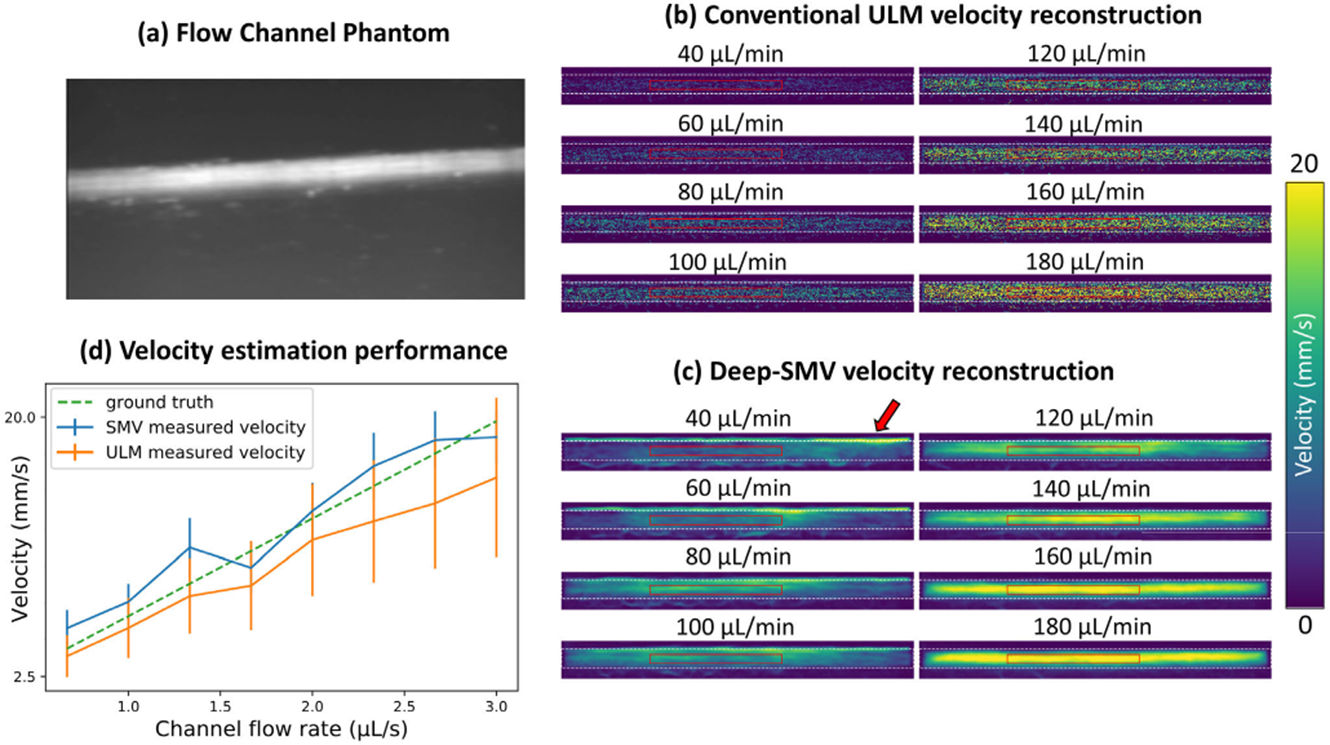
Flow channel phantom validation. (a) A contrast power Doppler image of the experimental flow phantom. (b) Conventional ULM localization and tracking was able to sparsely populate the flow channel with velocity estimates. (c) Deep-SMV was also able to provide velocity estimates for this flow channel and was much more efficient in using the MB signal, resulting in a higher proportion of reconstructed luminal space. (d) The performance of velocity estimates for different flow volume rates between ULM and Deep-SMV, in comparison to the theoretical gold-standard. The analysis ROIs were placed to avoid the upper edge enhancement artifact from buoyant MBs at low flow volume rates (arrow).

A pair of holes were drilled on the opposite walls of a clear rectangular plastic container. A stainless-steel rod with a diameter of 500μm was inserted horizontally into the container through the openings. A 20% gelatin mixture was poured into the container until the surface was approximately 5mm above the metal tube. The phantom was placed in a 4°C refrigerator until the gelatin completely solidified. The metal tube was removed prior to the experiment to create a flow channel, the diameter of which constricted to approximately 450μm during the gelatin solidification process. The inlet to the flow channel was connected to a programmable syringe pump (NE-300, New Era Pump Systems Inc., Farmingdale, NY) with a soft plastic tube to provide constant flow volume rate through the channel. A clinically available ultrasound contrast agent (DEFINITY^®^, Lantheus Medical Imaging, Inc.) was diluted 1000-fold with 0.9% saline (0.9 sodium chloride, BD, Franklin Lakes, NJ) and was perfused through the flow channel. Volume rates of 40−180μL/min with 20μL increment was used in this experiment. The L35-16vX transducer was placed at the top surface of the phantom and positioned to provide a longitudinal view of the flow channel (Fig. 4a). For each volume rate, we waited until a stable flow was formed before starting the acquisition of 1.6s duration (1,600 frames).

### F. Ex Ovo CAM Imaging

**Fig. 5.**
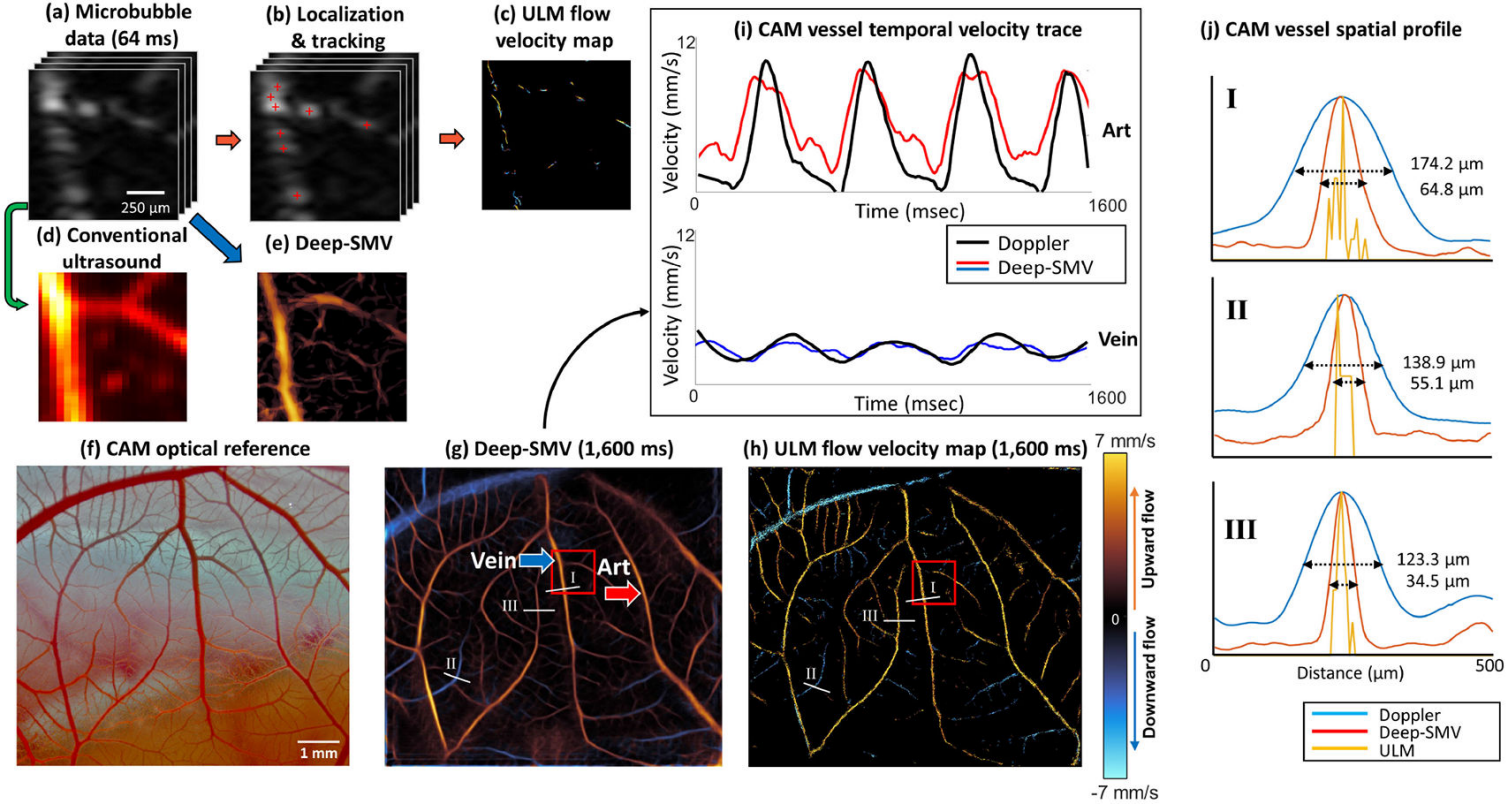
Deep-SMV workflow on CAM surface vessel data. (a) An example short data segment of MB data (64 ms) was processed using (b) conventional ULM localization and tracking, resulting in (c) a very sparse flow velocity map where it is difficult to visualize the microvessel anatomy. (d) Direct accumulation of MB signal data reveals diffraction-limited vessel structure. (e) Processing the same data segment with Deep-SMV allowed for super-resolution velocity estimation with more structural connectivity than conventional ULM. (f)-(h) Deep-SMV and ULM performance comparison on long data segments with large FOV. (i) Deep-SMV shows distinct pulsatility features in local regions sampled from feeding (Art) and draining (Vein) vasculature, which matched conventional Doppler velocity measurements. (j) Diameters of three different-sized vessels measured by the FWHM of power Doppler, Deep-SMV and ULM reconstructions.

**Fig. 6.**
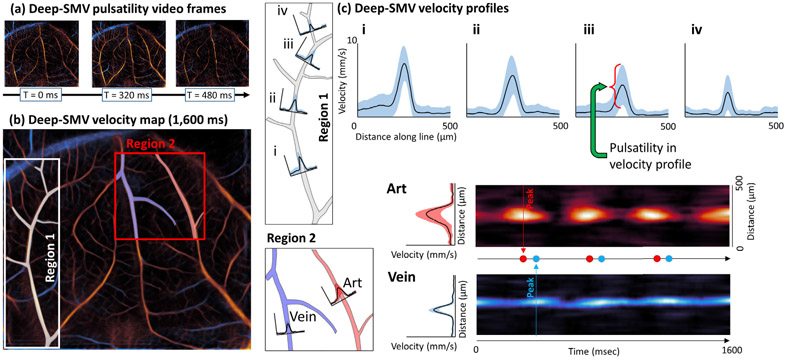
Detailed CAM flow dynamics analysis using Deep-SMV velocity estimates. (a) Video frames taken from a Deep-SMV pulsatility video which demonstrate cycling pulsatility and different flow dynamics for feeding and draining vessels. (b) Accumulated Deep-SMV velocity map with two selected regions. Region 1 demonstrates a gradual decrease in (c) peak velocity and pulsatility in velocity profiles along the vessel length (i to iv). Region 2 demonstrates a phase delay in the peak velocity estimate for vessels labeled Art and Vein.

The fertilized chicken egg incubation, preparation, and contrast injection were performed following the procedure described in [32] and [33]. The surface vasculature of the CAM was injected with 70 μL of DEFINITY^®^at clinical bolus concentration immediately prior to ultrasound imaging. The L35-16vX transducer was placed on the side of the plastic weigh boat to image the surface vasculature of the CAM (Fig. 5g, Fig. 6).

### G. Mouse Brain Imaging

**Fig. 7.**
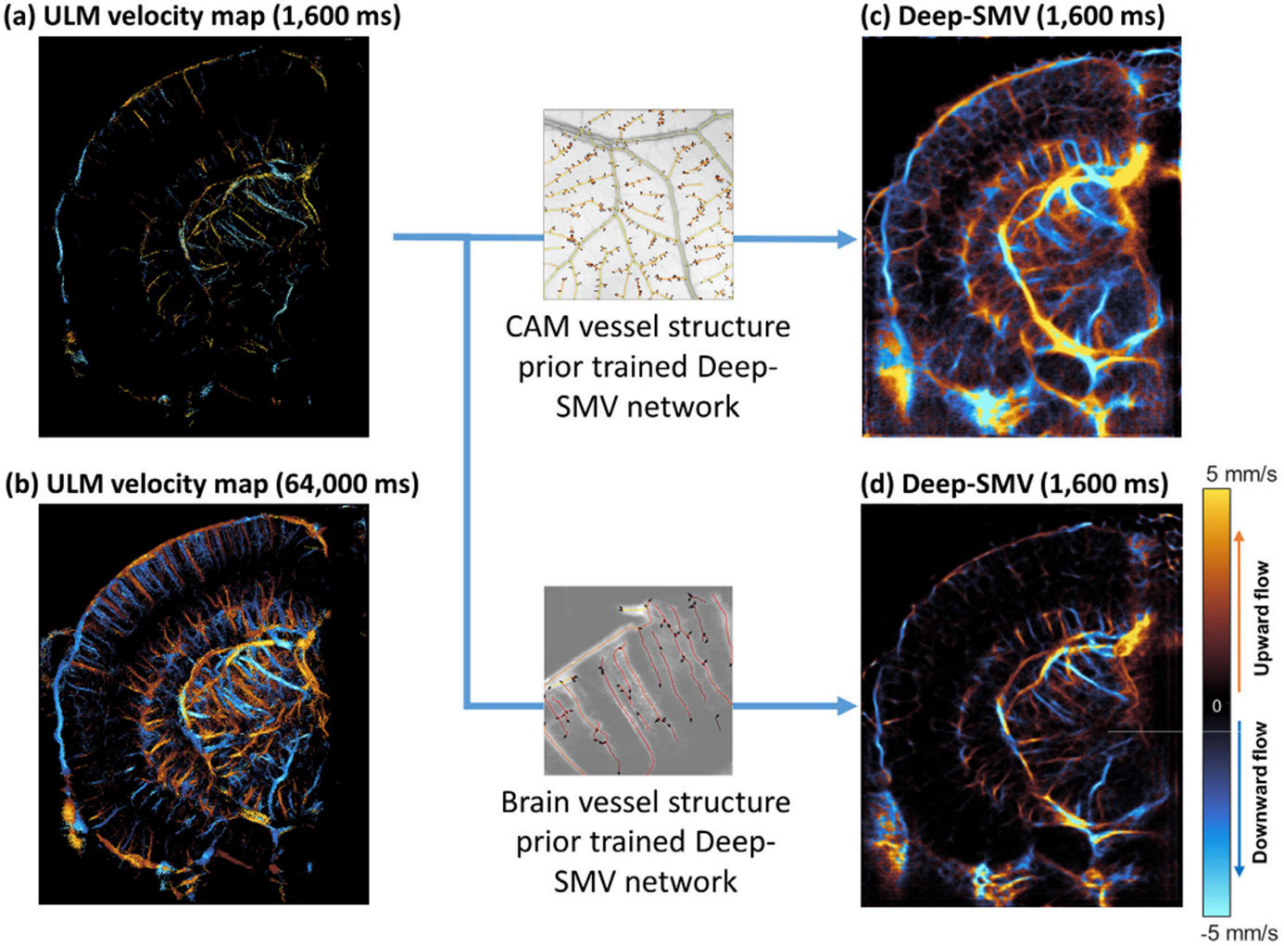
Deep-SMV with different vessel structure prior. (a) Conventional ULM localization and tracking for a 1600ms mouse brain dataset was only able to sparsely populate the image with velocity estimates. (b) Due to the inefficient use of MB signals, a much longer acquisition time (64,000ms) was required to reconstruct the majority of brain vasculature. (c) Deep-SMV trained with CAM simulation data had acceptable performance for larger subcortical vessels but was unable to separate parallel cortical vessels. (d) The mouse-brain prior trained Deep-SMV network had improved performance in the cortical regions, while maintaining the ability to reconstruct subcortical features.

**Fig. 8.**
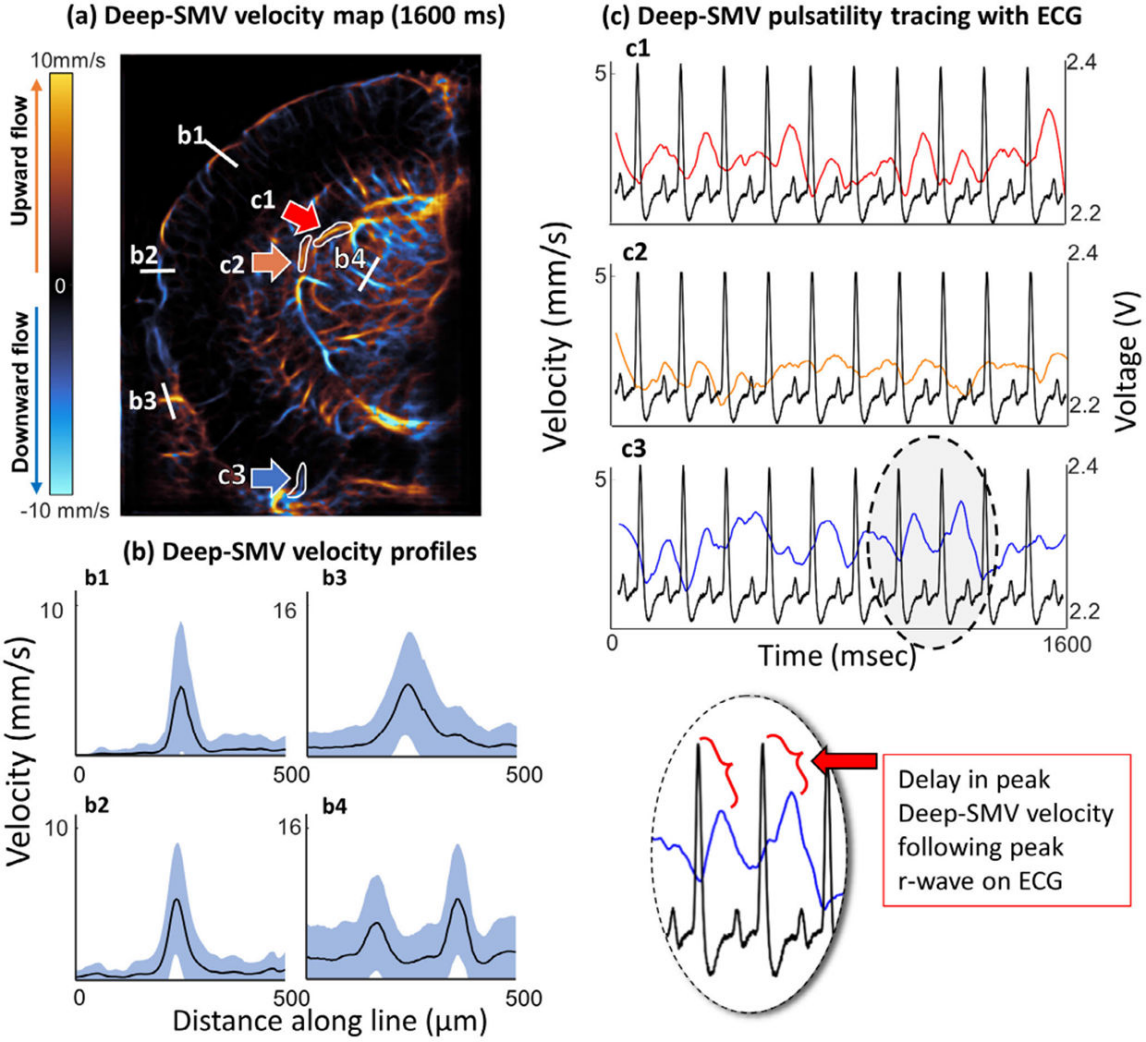
Mouse brain vessel velocimetry validated with ECG. (a) Deep-SMV velocimetry map of mouse brain. (b) Velocity profiles generated from cortical (b1 and b2) and subcortical (b3 and b4) vasculature. (c) Velocity traces of cortical vessel (c1) and subcortical vessel (c2 and c3) segmentations in comparison to ECG measurements.

Anesthesia, craniotomy preparation, and animal handling were performed using the protocol described in [34]. The L35-16vX transducer was positioned to acquire a coronel imaging plane of the brain through the cranial window (Fig. 7, Fig. 8). Fresh DEFINITY^®^was activated and mixed with sterile saline to achieve a concentration half of the diluted clinical bolus guideline (0.65 mL DEFINITY^®^with 8.7mL saline). A 50 μL bolus injection of contrast was perfused via the tail vein catheter to confirm vessel patency, and two 1,600 frame data sets were acquired at this relatively high MB concentration. A continuous infusion of contrast was then performed at a rate of 10 μL/min using a programmable syringe pump, as per [35]. The MB solution was mixed every 5 minutes using a magnetic stirrer to maintain a constant MB concentration during the experiment. Once a steady-state MB perfusion was confirmed, a total of 40 acquisitions (64,000 frames, or 64 seconds of data) were acquired for this imaging plane. Electrocardiogram (ECG) signal was acquired using an iWorx (Dover, NH, USA) IA-100B single channel biopotential amplifier with C-MXLR-PN3 platinum needle electrodes inserted into the legs of the animal.

### H. Conventional ULM Image Processing and Analysis

MBs were extracted for each ultrasound IQ dataset by applying a spatiotemporal singular value decomposition (SVD)-based clutter filter [5], [21], [32], [36], where the low-order singular value threshold representing tissue signal was determined adaptively [37]. A noise-equalization profile was applied to account for depth-dependent effects [38]. An isolated MB signal was manually identified and fit to a multivariate Gaussian function to represent the point-spread function (PSF) of the system. A MB separation filter [21] was applied to each SVD-filtered dataset, and then the imaging data was spatially interpolated to an isotropic 4.9 μm axial/lateral resolution using spline interpolation [39]. MBs were then localized by applying a 2D normalized cross-correlation between the empirical PSF and each frame of interpolated IQ data. MB centroid pairing and frame-to-frame trajectory estimation was performed using the uTrack algorithm [40], or a Kalman filtering algorithm [41], where velocity was calculated from centroid displacement. Each processed data acquisition was accumulated into a final reconstruction.

## III. Results

### A. Deep-SMV Architecture Efficiently Uses Spatiotemporal MB Data

An example of in vivo informed data simulation for Deep-SMV training is demonstrated in Fig. 3. CAM optical images (Fig. 3a) provide a physiologically relevant in vivo vascular structure that can be readily segmented (Fig. 3b) to generate binarized representations of the vessel space. The process of medial axis skeletonization can then yield structural connectivity maps (Fig. 3c) as well as distance transform maps of this vessel bed, which is informative of the local vascular diameter. This serves as the basis for an undirected vessel graph for MB flow simulation (Fig. 3d), which is assigned direction using a breadth-first search (BFS) traversal algorithm (Fig. 3e). The resulting MB locations were fed into a Field-II [30], [31] simulation (see Fig. 3f, detailed in Methods) to generate training samples of 16 frames in the temporal dimension, and 256 * 256 pixels in the spatial dimension.

### B. Flow Channel Phantom Validates Deep-SMV Reconstructions

We then tested the performance of Deep-SMV on an in vitro flow channel phantom (Fig. 4) where the ground-truth velocity is known from the flow volume rate of the connected syringe pump. A contrast power image of the flow channel (Fig. 4a) demonstrates that the flow channel was well perfused with MB contrast agent, yielding a high MB concentration scenario where conventional ULM does not perform efficiently. This is apparent in the conventional ULM velocity reconstructions (Fig. 4b) where MB localization and tracking results in relatively sparse tracing of the flow channel lumen. Deep-SMV was also used to process the same dataset (Fig. 4c). Deep-SMV demonstrated a better realization of the parabolic flow profile within the flow channel phantom with a much higher perfusion efficiency. In comparison to the mass-conservation calculated ground-truth (Fig. 4d), conventional ULM showed an underestimation bias for velocity estimates, particularly for high flow volume rates, whereas Deep-SMV was more consistent across all tested flow velocities. The analysis regions of interest (ROIs) were placed to avoid the upper edge enhancement artifact from buoyant MBs at low flow volume rates (arrow).

### C. Deep-SMV Outperforms Conventional ULM on Short Data Segments

The use of Deep-SMV for the generation of super-resolved velocity maps takes advantage of the fast forward-processing speed of NNs and GPU-based parallelized computing to achieve real-time super-resolution imaging capability. The example 300 x 300-pixel image patch with 64 temporal frames in Fig. 5a took approximately 2,500 ms to undergo the conventional ULM localization and tracking steps (Fig. 5b) to produce the sparsely populated super-resolution flow velocity map shown in Fig. 5c. The direct temporal accumulation of MB signals, a conventional diffraction-limited ultrasound technique shown in Fig. 5d, demonstrates that most of the vascular luminal space is missed in conventional ULM for a short data segment due to the inefficient MB localization process. Applying Deep-SMV to the same data segment yielded a super-resolution flow velocity map (Fig. 5e) which only took 28 ms to process on 2 NVIDIA^®^GeForce^®^RTX 2080 Ti GPUs, a near 100X acceleration compared to conventional ULM. The magnitude of flow estimated via Deep-SMV is comparable to the point estimates derived from ULM; however, it is notable that the proportion of vascular lumen space that was reconstructed is much higher than conventional processing. The convention used in all results presented is that the orange color indicates flow toward the transducer, and blue indicates flow away from the transducer.

### D. Chicken Embryo CAM Velocimetry Reveals Super-Resolved Vascular Pulsatility

After demonstrating good performance in the flow phantom experiment (Fig. 4) and for small in vivo imaging patches (Fig. 5a-e), we then sought to test Deep-SMV on longer duration in vivo contrast ultrasound data from a large imaging field of view (FOV). We have previously shown that the CAM is an excellent model for testing ultrasound super-resolution processing methods [21], [41], [42], providing low attenuation and minimal tissue motion, while also being accessible to optical imaging to serve as a reference standard (Fig. 5f). Deep-SMV processing of a 1600-frame (1,600 ms) IQ data acquisition (Fig. 5g) reveals an interdigitated vessel structure that is characteristic of the CAM vascular topology, and which shows good correspondence to the optical reference. The small branching arterioles on the order of 20-30 μm in diameter, which are adjacent to the main site of gas exchange, are visible in the Deep-SMV reconstruction of this planar vascular membrane. The conventional ULM reconstruction of this same dataset (Fig. 5h) shows comparable performance for larger vessels but was less able to distinguish the smaller interdigitated vasculature on the CAM than Deep-SMV due to the relatively short data acquisition time. To provide validation of this spatial resolution gain achieved by Deep-SMV, we selected three different sized vessels (marked in Fig. 5g) to plot the vessel cross-sections of conventional power Doppler, Deep-SMV, and ULM reconstruction using 1.6s of ultrasound data (Fig. 5j). The resolution was quantified by the full width at half maximum (FWHM) values of the vessels. In the larger sized vessel shown in Fig. 5j**-I**, Deep-SMV reconstruction achieved a 2.8x resolution gain as measured by FWHM in comparison to power Doppler (64.8 μm vs. 174.2 μm, respectively). The resolution gain was increased to 3.6x for the smallest sized vessel in Fig. 5j**-III** (34.5 μm vs. 123.3 μm, for Deep-SMV vs power Doppler). Conventional ULM produced the thinnest vessel reconstruction. However, it was also the noisiest within vascularized regions due to the short accumulation time.

In addition, Deep-SMV had the added advantage of providing a higher temporal resolution by enabling satisfactory super-resolution reconstruction within a short period of time (e.g. 160 ms), by exploiting the relatively high MB concentration in this dataset. As demonstrated in Fig. 5i, Deep-SMV revealed a cyclic pulsatility in the blood flow velocity within two selected vessel regions (labelled **Art** and **Vein**) that were not apparent in conventional ULM processing. The frequency of the change in blood velocity was around 2-3 Hz, which matches with the Doppler fluctuation, and is close to the reported values for the cardiac cycle of chicken embryos at this stage of development [43].

To further investigate the utility of Deep-SMV for super-resolved pulsatility mapping, we performed a detailed CAM vessel flow dynamics analysis in Fig. 6. Fig. 6a demonstrates selected imaging frames (160 ms between each frame) from a Deep-SMV pulsatility video.^1^ At the beginning of the cardiac cycle in the chicken embryo, the main feeding vessels to this vascular bed accelerate to a peak velocity and demonstrate a rapid cyclic shift in the Deep-SMV velocity estimates over time. In contrast, the draining vessels show a much slower peak velocity, with less dramatic cycling and a slight phase delay in peak velocity time. Fig. 6b demonstrates a local analysis performed on two regions within the accumulated Deep-SMV velocity map. In **Region 1**, we note that there is a gradual decrease in the peak velocity and pulsatility in the velocity profiles (Fig. 6c) that were generated along the vessel length Fig. 6c **i-iv**). **Region 2** demonstrates the difference in peak velocity for the vessel cross-sections labeled **Art** and **Vein**, with a phase delay in the peak velocity estimate time apparent in the heat maps demonstrated, implying that MB flow needed time to cross the capillary space between these two vessels. These observations are consistent with literature investigating pulsatile variation in the CAM model with optical imaging [44].

### E. Tissue-Specific Training Data Improves the Performance of Deep-SMV

Next, we applied Deep-SMV to the extremely complex vascular structure that is present in the mouse brain. The densely packed and hierarchical vascular organization of the mouse brain presents a substantial challenge for super-resolution vascular imaging, typically requiring very low concentration MB injections and prohibitively long data accumulation times to accurately reconstruct cerebral features. A case point for this difficulty is evident in the example shown in Fig. 7a, a short duration (1,600 ms) ULM reconstruction of the brain vasculature. This example exemplifies the graduality of ULM accumulation, with the sparsity of the reconstruction evident in both the columnar cortical vessels and the sub-cortical supply vasculature. Only after a substantially longer accumulation time does mouse brain hierarchical vasculature structure become apparent in conventional ULM reconstruction (Fig. 7b – requiring 64,000 ms of data). Sporadic MB localization errors are also evident, particularly in the largest vessels, leading to spurious velocity estimates which bias the final velocity map. In comparison, the Deep-SMV network used in the previous section was used to reconstruct a velocity map using only 1,600 ms of data (Fig. 7c), which demonstrates multiple scales of vasculature, with obvious branching connectivity ranging from the interior feeding arterioles to the cortical capillary layers – highlighting Deep-SMV’s more efficient use of high concentration MB signal.

In order to study the effect of a structural prior in the training data, we re-trained the model from scratch using a mouse brain ULM based simulation training set and applied it to mouse brain data. While the CAM-trained network has acceptable performance on larger vessels (Fig. 7c), it was unable to separate parallel cortical vessels because such structure is rarely seen in the CAM template, where vessels are more evenly spaced and smaller-sized vessels rarely exhibit densely packed three-dimensional patterns. Using mouse brain ULM trained Deep-SMV model, most of the cortical vessels can be resolved with improved clarity (Fig. 7d). We thus concluded that the performance of Deep-SMV is indeed affected by the structural bias in the training data. Similar performance gain may be possible using training simulation from mouse brain optical imaging, such as in [19], ex vivo micro-CT as in [45], or from publicly available histological atlases. However, we note that in cases where a vascular model of the organ of interest cannot be easily obtained, using training data from a different organ is still a feasible option as long as the scales of the vasculature are comparable.

A final comparison of super-resolution imaging performance in demonstrated in Table II. Fourier ring correlation (FRC) was performed on ULM reconstructions to estimate the spatial resolution of reconstruction, as per the randomized track splitting strategy proposed by Hingot et al. [46]. FRC analysis was also performed on Deep-SMV vessel reconstructions by temporally accumulating alternating non-overlapped frames to produce two independent reconstructions. However, this may result in uneven estimates of velocity due to uneven sampling of the cardiac cycle, so a normalized approach was used to accumulate detected flow events – an analogue to MB localization events in conventional ULM reconstruction. Both ULM and Deep-SMV were found to have an estimated spatial resolution below a half wavelength for our 20MHz imaging frequency (38 μm).

### F. Mouse Brain Deep-SMV Pulsatility Follow Cardiac Cycle

Motivated by Deep-SMV’s ability to visualize super-resolved vascular pulsatility in the CAM (Fig. 6), we applied a similar flow dynamics analysis to a Deep-SMV reconstruction of mouse brain vasculature (Fig. 8). Velocity line profiles taken from cortical arterioles (Fig. 8a) demonstrated a well-developed laminar profile with cyclic pulsatility. Like-wise, velocity line profiles could also be generated for deep sub-cortical vessels, such as in and around the thalamus. Segmentation ROIs were placed around cortical and sub-cortical vessels (Fig. 8b) to generate velocity traces over the course of the 1,600 ms imaging acquisition. The pulsatility of the Deep-SMV velocity estimates (Fig. 8c) matched electrocardiogram (ECG) measurements taken from this mouse, with a slight shift in phase demonstrated between the peak velocity and ECG peak. In the entorhinal cortex, the ECG r-wave peak preceded the peak velocity measurement by an average (± standard deviation) of 85.8 ± 24.7 ms. For the subcortical regions, the dorsal vessel reached peak velocity 99.0 ± 17.9 ms after the ECG r-wave peaks, and the lateral flowing vessel had the shortest delay in peak velocity at 68.1 ± 28.4 ms.

## IV. Discussion

In this study, we introduced a DL-based super-resolution microvessel velocimetry approach for contrast-enhanced ultrasound imaging, named Deep-SMV, by employing LSTM blocks in the bottleneck layers of a UNet architecture to take advantage of the rich spatiotemporal information that is present in ultrafast ultrasound MB data. Using this architecture, we can bypass the extremely expensive MB localization and tracking processing in conventional ULM to provide super-resolved blood velocity measurements that are more robust to high MB concentrations and at a high imaging speed. The training data for the Deep-SMV network was generated using ultrasound MB flow simulation on in vivo optical images taken from the CAM of chicken embryos and mouse brain ULM images. The technique was validated using a flow channel phantom, where less velocity underestimation bias, in comparison to ULM, was noted for fast flow rates. This is possibly due to Deep-SMV avoiding the explicit MB frame-to-frame linking data association step, which may lead to spurious trajectory reconstructions. We also demonstrated that Deep-SMV can produce super-resolved velocity maps for several challenging in vivo imaging scenarios, including chicken embryo CAM imaging and mouse brain imaging.

Previous research groups have applied CNNs to the problem of extracting useable ULM localization data from overlapping MB signals [16], [17], [18], [19]; however, none of these approaches yield velocity information. While it is possible to perform inference on spatiotemporal data with a conventional CNN by treating the temporal dimension as multiple channels or as an additional spatial dimension, the LSTM-based spatiotemporal model has several advantages over conventional CNN approaches. The scale of temporal perception of a multi-channel CNN or 3D CNN is limited by the number of frames in a single input sequence, which is often subject to memory constraints. Longer sequences need to be partitioned into shorter segments to accommodate the CNN input size. The CNN will not form any connection between different input segments, which means that it can only detect local temporal features. LSTM, on the other hand, can handle arbitrary length inputs. With the help of internal hidden states, LSTM can recall information obtained from previous inputs for a long time, making it more suited for the task of velocity estimation.

We also demonstrated that the Deep-SMV approach substantially improves super-resolution reconstruction speed, particularly for regions with high vessel densities, allowing for real-time visualization of the microvascular flow dynamics. This is a particularly enticing accomplishment for super-resolution ultrasound imaging, potentially enabling the translation of the technology into functional ultrasound applications where high temporal resolution is critical for monitoring vascular response(s). Although other DL-based approaches have demonstrated rapid localization times, these solutions still rely on conventional ULM pairing and tracking algorithms to estimate velocity. The processing time burden of these algorithms scale with the MB count, making these solutions ineligible for dynamic real-time velocimetry applications. The Deep-SMV network processing time does not depend on the MB concentration, performing consistently across multiple imaging scenarios. However, applying Deep-SMV to MB data with too-low concentration will lead to sparse and noisy reconstruction, due to insufficient information available for reconstruction. Slight underestimation may also be present due to temporal averaging of a sparser estimation. Moreover, since Deep-SMV is trained to produce velocity estimation in pixel per frame, it can be easily adapted for different imaging framerates by adjusting the conversion from pixel-space unit to physical units. The training set would also need to be adjusted to represent the appropriate physical velocity range for accurate results.

Although the Deep-SMV approach shows substantial promise over conventional ULM and competing DL solutions, the results presented in this manuscript must be understood within the context of the limitations of the study. The most critical is that there is no established gold-standard technique for measuring microvascular flow velocity to validate the output of the Deep-SMV network. As a compromise, we have used the velocity results of the much more computationally expensive ULM reconstructions as a reference standard; however, this approach comes with some caveats. As discussed, ULM processing is susceptible to spurious MB localizations which may bias the velocity point estimates. We have attempted to account for this by averaging the ULM velocity references across a long acquisition time, which should reduce the impact of this source of error. The network was trained using Field II simulations of MB flow, where the velocity distribution was taken from experimental ULM reconstructions of vasculature. This implicitly tunes some aspects of the Deep-SMV network to the parameters used in the initial ULM reconstruction and is also dependent on the assumptions applied during vascular modeling (e.g., laminar flow). This is demonstrated directly by the gain in Deep-SMV performance for organ-specific simulation data in comparison to more generic vascular flow training (Fig. 7). However, it is important to note that the use of organ-specific training data will result in the network having an inherent structural bias. In regions where solving the underlying blood vasculature and flow dynamics is ill-posed, such as where vessels are intertwined, very closely distributed, or where MB signal is very sparse (i.e., only one event within a vessel segment in hundreds of frames), the model will attempt to generate estimations based on insufficient information, which can appear as artifact vessel structures that did not exist in the original data. The distributions of these hallucinated vessels will most likely reflect the typical distribution of vessels present in the training set. The misleading fake vessels could become a potential risk for practical applications of Deep-SMV. A potential approach to minimize this risk is to employ a constraining factor that is equivalent to persistence control in many of the conventional ULM tracking methods, where signals are deemed unreliable and their associated estimation results are discarded. Field II also cannot simulate nonlinear MB responses. Furthermore, although Deep-SMV demonstrates better handling of high MB concentrations, it is still susceptible to signal interference of densely packed MBs leading to a retrograde flow aliasing artifact in some larger vessels. The incorrect flow direction is also apparent in ULM reconstructions, but the long acquisition duration can reduce this effect.

At present, the flow dynamics modeling we assumed for simulation is relatively simplistic. Employing more advanced features, such as dynamic velocity profile in the vessel cross-section, diameter-dependent viscosity, and pulsatility within a simulated data segment, can potentially improve the robustness of Deep-SMV for complex blood flow patterns. Physiologically relevant constraints in assigning flow velocity and connectivity can also be implemented.

In the current training setup, we used MSE loss, which is a loss function based on the Euclidean distance between the target and the estimation. Although MSE has been successfully applied to various image processing tasks, it is not necessarily the most efficient in capturing errors in our desired output data. We believe that a loss function specifically tailored for velocimetry can potentially improve the performance of Deep-SMV.

Another loss term that we considered was the structural similarity (SSIM) loss, a loss designed to provide a similarity measurement between two images that reflects how humans perceive them. Since structural similarity is normally applied to images scaled to a uniform range (0-1 or 0-255), using it on its own is not well-suited for our task, where the velocity range can be arbitrary. Instead, we experimented with a combined loss function with MSE as the dominant loss term and SSIM as an additional constraint on vascular structure. We discovered that the addition of SSIM term did not improve the quality of reconstructed vessel structure. This might be due to the fact that perception of structure in natural images is different from that in microvessel images. Small distortions that normally do not heavily affect the image quality of a natural image, and thus penalized less when using SSIM loss, can have greater impact in preserving vascular structure.

Deep-SMV provides efficient, effective, and fast super-resolution microvascular structural and velocity reconstruction without requiring MB localization, pairing, and tracking. By eliminating this inefficient ULM processing pipeline, Deep-SMV can capitalize on the rich spatiotemporal information present in the input IQ data, enabling real-time super-resolution imaging that is robust to a wide range of imaging conditions. This technique demonstrated a high temporal resolution, permitting the application of super-resolution velocimetry to functional imaging scenarios and enabling super-resolved pulsatility mapping. By relaxing the strict imaging paradigm that ULM typically requires, Deep-SMV can facilitate the translation of super-resolution ultrasound to a wider range of experimental and clinical applications.

## Supplementary Material

supp1-3251197

## Figures and Tables

**TABLE I:** Field-II Simulation Parameters

Variable Name	Value
Transducer type	Linear array
Number of transmit elements	128
Number of receive elements	128
Element height	80 μm
Element width	62 μm
Pitch	70 μm
Transmit apodization	Kaiser
Receive apodization	Rectangle
Center frequency	20 MHz
Sampling frequency	125 MHz
Speed of sound	1540 m/s
Number of compounding angles	9
Transducer type	Linear array

**TABLE II:** Fourier Ring Correlation Resolution Estimates

	CAM ½ bit	CAM 2-σ	Mouse brain ½ bit	Mouse brain 2-σ
ULM	27.4 μm	22.8 μm	24.4 μm	19.4 μm
Deep-SMV	41.0 μm	27.2 μm	54.7 μm	33.8 μm

## Footnotes

1 Supplementary video available in the supporting documents /multimedia tab.
